# Diaphragmatic perforation with colonic herniation due to hepatic radiofrequency ablation: A case report and review of the literature

**DOI:** 10.3892/ol.2013.1625

**Published:** 2013-10-15

**Authors:** MEIQI ZHOU, HAIFEI HE, HONGKE CAI, HAILONG CHEN, YUE HU, ZHENG SHU, YONGCHUAN DENG

**Affiliations:** Department of Surgical Oncology, Second Affiliated Hospital, Zhejiang University College of Medicine, Hangzhou, Zhejiang 310009, P.R. China

**Keywords:** radiofrequency ablation, hepatocellular carcinoma, diaphragmatic perforation, hernia, major complication

## Abstract

Radiofrequency ablation (RFA) has been widely accepted as an alternative treatment for unresectable primary and metastatic hepatic tumors, with satisfactory rates of local response and significant improvements in rates of overall survival. Numerous large series studies have shown that RFA is safe and effective, with a low mortality rate and a low major complication rate. Major complications, including diaphragmatic perforation and hernia, have rarely been previously reported. The current case report presents a case of diaphragmatic hernia with perforation of the incarcerated colon in the thoracic cavity 12 months following hepatic RFA, and reviews nine previously reported cases of diaphragmatic hernia. Comprehensive analysis of the nine cases demonstrated possibilities leading to diaphragmatic hernia following diaphragmatic thermal injury as a consequence of hepatic RFA. Clinicians and radiologists must consider diaphragmatic thermal damage following hepatic RFA for liver tumors adjacent to the diaphragm, particularly for patients with symptoms of ileus, dyspnea, chest pain, pleural effusion and right shoulder pain.

## Introduction

Radiofrequency ablation (RFA) has been widely accepted as an alternative treatment for unresectable primary and metastatic hepatic tumors (1), as it achieves a satisfactory local response rate and significantly improves overall survival rates compared with other modalities, including chemotherapy or percutaneous ethanol injection (2). However, for patients with small or solitary hepatic tumors (primary or metastatic), RFA is inferior to hepatic resectioning with a reduced survival benefit (3–5). Numerous large series studies have shown that RFA is safe, with a low mortality rate (0–2%) and a low major complication rate (6–9%) (2,3).

According to recommendations proposed by the Working Group on Image-Guided Tumor Ablation, major complications of RFA, including hollow viscera perforation (0.3%) (6), biliary stenosis and skin burns (3,7), are life threatening if left untreated, and likely to lead to substantial morbidity and disability, hospital admission or substantially longer hospitalization. Major complications, including diaphragmatic perforation and hernia, have rarely been previously reported. When the target tumor abuts the diaphragm, the risk of diaphragmatic thermal injury increases, as the surgeons are unable to dissect the dome of the liver from the diaphragm, and overlapping ribs and lungs obscure ultrasonic visualization. The current case report presents a case of diaphragmatic hernia with perforation of the incarcerated colon in the thoracic cavity 12 months following hepatic RFA. Written informed consent was obtained from the patient.

## Case report

**Figure 1:**
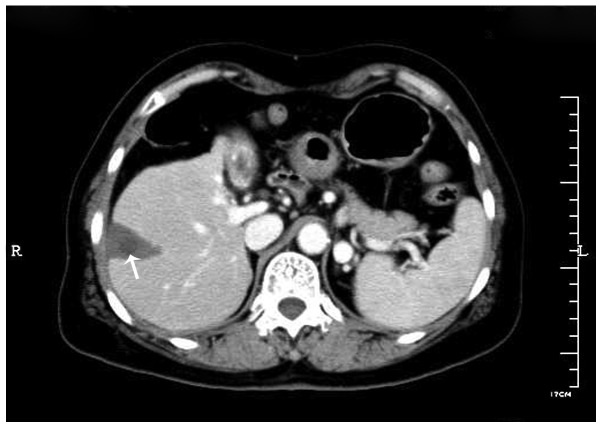
Abdominal CT image following hepatic RFA shows a wedge-shaped lesion of ablation extending to the outer edge of segment VIII in the liver. A low-density lesion 1.5 cm in diameter represents the location of the hepatic tumor, as indicated by the white arrow. CT, computed tomography; RFA, radiofrequency ablation.

A 61-year-old female was admitted to the Second Affiliated Hospital of Zhejiang University College of Medicine (Hangzhou, China) on December 11, 2010, complaining of lower abdominal pain with nausea, vomiting and constipation. The patient had a medical history of hypertension, coronary heart disease, hepatitis B, cirrhosis and hepatic RFA for hepatocellular cancer in segment VIII of the liver 12 months prior to admission (Fig. 1). During the intervening 12 months, there was no history of trauma or surgery. An abdominal X-ray showed an elevation of the right hemidiaphragm and an air-fluid level in the subphrenic intestine. The patient was initially diagnosed with an ileus of unknown cause and was managed conservatively.

**Figure 2:**
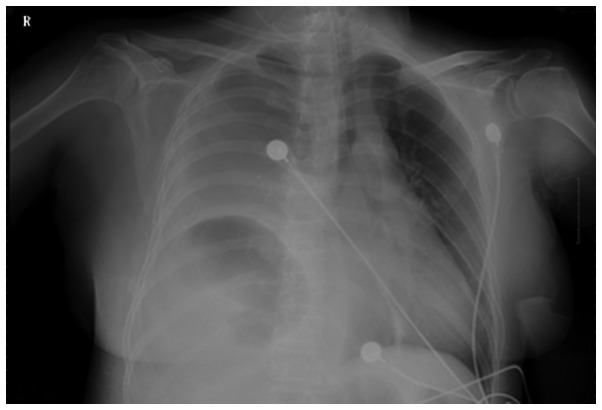
Chest X-ray shows a right pleural effusion and enlarged bowel in the chest cavity.

**Figure 3:**
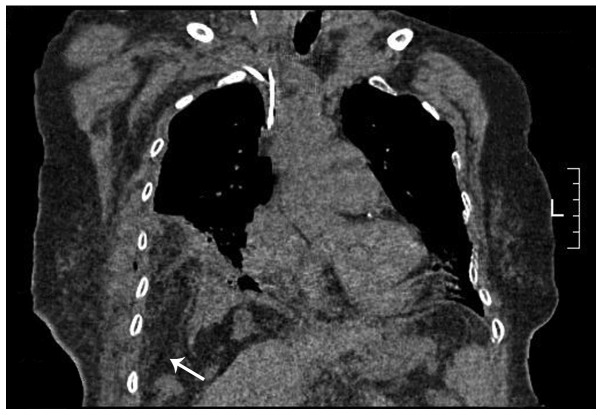
A coronal thoracic CT image shows a right diaphragmatic defect and loops of the bowel protruding into the thoracic cavity, as indicated by the white arrow. CT, computed tomography.

Ten days later, the patient developed respiratory failure and shock with an onset of acute chest pain and high fever. The patient was transferred to the ICU for mechanical ventilation and life support. An emergency chest X-ray revealed a right pleural effusion and enlarged bowel in the chest cavity (Fig. 2). Diaphragmatic defect was visualized by coronal thoracic computed tomography (CT) imaging (Fig. 3). Closed drainage of the pleural cavity and antibiotic treatment were administered. Feculent fluid was drained through a chest tube, indicating that the patient suffered from a diaphragmatic hernia with incarcerated colon perforation and pyothorax. Emergency laparotomy was performed and showed a section of necrotic transverse colon with perforation and a large quantity of pus in the pleural cavity. It is likely that infarction and perforation occurred following colon herniation into the pleural cavity. Following the return of the herniated colon to the abdomen, a defect in the diaphragm measuring 4 cm in diameter was found abutting the liver. A spot of thermal focal damage was located beneath the defect, at the position of the previous RFA treatment for hepatocellular carcinoma. The perforated diaphragm was not adherent to the liver, so direct tumor invasion may be excluded.

In addition, intestinal necrosis was identified 70 cm from the ileocecal valve and the proximal small intestine was enlarged with gas accumulation. A transverse colectomy with proximal colostomy, ileum resection with side-to-side anastomosis, thoracic irrigation and simple suture of the diaphragmatic defect were completed. The patient recovered well and was discharged from hospital two months following surgery. Written informed consent was obtained from the patient for publication of this case report and the accompanying images.

## Discussion

A diaphragmatic hernia is the protrusion of abdominal structures into the thorax as a result of congenital, traumatic and iatrogenic defects in the diaphragm. Iatrogenic defects are not common and may be associated with RFA or abdominal surgery for organs adjacent to the diaphragm, including the liver, lung or spleen. Diaphragmatic perforation and herniation are rare major complications of hepatic RFA. Systematic studies evaluating the efficacy and safety of RFA for hepatocellular carcinoma adjacent to the diaphragm or in other hypothesized high-risk locations have not been described (8–13) and only eight case reports were found in the previous literature (14–21). The present case report reviewed a total of nine reported cases (including the current case) of diaphragmatic hernia following hepatic RFA (Table I).

Among the nine cases listed in Table I, the age at diagnosis ranged between 37–72 years (mean, 56.9 years) and there were five females and four males. The majority of the primary hepatic tumors were single lesions measuring ~2 cm in diameter, with the exception of one tumor of >5 cm (16) in diameter and multiple lesions were involved in two cases (14,20). Common locations of targeted lesions were the superior segments in the right hepatic lobe (S8, S7 and S5) adjacent to the diaphragm. Treatment details of RFA were not available for every case and in two cases (14,15), expandable hook-shaped electrodes were described. Eight cases were performed under ultrasound guidance and one case was under CT (21). Two cases (15,19) underwent repeated RFA, with interval times between hepatic RFA and diaphragmatic herniation ranging between 5–20 months (mean, 13.3 months). The amount of heat delivered to the tumors may be an additional risk factor, but there have been only two reports of the use of a peak power of up to 75 (14) and 120 W (21). Early symptoms of ileus, including nausea, vomiting and intermittent abdominal pain, were present for a prolonged time in all nine cases and acute chest pain or dyspnea occurred when abdominal structures suddenly herniated into the pleural cavity or incarcerated bowel rupture. Only one patient received conservative treatment without surgery. The remaining eight patients recovered well following surgical repair (three cases by laparoscopy) of the diaphragmatic defect with or without colectomy, and one patient succumbed to hepatic tumor rupture one month following surgery (14). In general, two of the nine (22.2%) cases of diaphragmatic hernia resulted in perforation of the incarcerated colon, respiratory failure or shock, requiring intensive care treatment followed by emergency colectomy (19).

Comprehensive analysis of the nine cases demonstrated specific possibilities leading to diaphragmatic hernia pursuing diaphragmatic thermal injury following hepatic RFA: Tumor adjacent to the diaphragm, poor liver function and hepatic cirrhosis, the use of an expandable type of RFA needle and the inability to confirm the position of the electrodes, pleural effusion and other complications with elevated abdominal pressure, including ascites, ileus and interposition of the intestine between the liver and diaphragm (Chilaiditi’s sign). The main mechanism of diaphragmatic hernia associated with RFA is diaphragmatic injury secondary to thermal or mechanical damage by the needle itself. The complication is particularly possible if the tumor abuts the diaphragm (13,14). Mechanical damage caused by the needle may lead to immediate perforation and thermal damage usually results in an inflammatory response leading to fibrosis, which ultimately weakens muscle fibers of the diaphragm and causes the defect (20). In addition, poor liver function may prevent the injured tissue from healing adequately. Complications of hepatic cirrhosis, including ascites and pleural effusion, may also promote tissue damage (14). The mechanism by which the colon migrates between the liver and diaphragm (Chilaiditi’s sign) has not been identified with certainty, but it may occur in patients with a redundant colon, chronic lung disease (including emphysema) or liver problems (including cirrhosis and ascites). Chilaiditi’s sign is generally not associated with symptoms and is most commonly identified incidentally in normal individuals. However, when diaphragmatic thermal injury is accompanied with Chilaiditi’s sign, the latter may facilitate the diagnosis of diaphragmatic perforation, which may lead to colonic herniation and strangulation.

To prevent or minimize diaphragmatic thermal injury, subphrenic artificial ascites (22) or intraabdominal carbon dioxide insufflation (23) have been indicated as simple and effective methods to separate the tumor from the diaphragm and to facilitate ultrasonic visualization, without a clinically confirmed heat-sink effect (1,22,24).

The early diagnosis of diaphragmatic perforation following hepatic RFA is often difficult, due to the lack of sensitivity and specificity of radiographic examination. Furthermore, diaphragmatic damage or a small perforation with adherence to the liver may be asymptomatic. Early symptoms, including nausea, vomiting and chronic abdominal complaints are often observed (8,16). Nevertheless, diaphragmatic perforation may remain unidentified unless there is visualization of abdominal contents herniated into the thoracic cavity and complicated by ileus with onset of acute dyspnea or chest pain (19). Elevation of serum markers, including CPK, LDH isoenzymes and AST, may be of value in identifying diaphragmatic muscle damage. A diaphragmatic hernia may be managed by thoracotomy or laparotomy (8). The diaphragmatic defect must be repaired and reinforced by a prosthetic mesh or closed only by suturing (8,15,16,18). If there is necrosis or perforation of incarcerated bowel, bowel resection is required and antibiotic treatment of pyothorax must be initiated.

In conclusion, this case report described a case of diaphragmatic hernia with perforation of the incarcerated colon in the thoracic cavity 12 months following hepatic RFA, and reviewed nine previously reported cases of diaphragmatic hernia. The early diagnosis of diaphragmatic perforation or hernia following hepatic RFA is often difficult. Clinicians must be aware of diaphragmatic thermal damage following hepatic RFA for liver tumors adjacent to the diaphragm, particularly for patients with symptoms of ileus, dyspnea, chest pain, pleural effusion and right shoulder pain. Radiologists must be aware of the integrity of the diaphragm to achieve early diagnosis of diaphragmatic perforation or hernia. We suggest that surgical repair must be performed once the diaphragmatic defect has been identified in order to avoid a diaphragmatic hernia complicated with acute intestinal obstruction or perforation, which may be lethal without immediate treatment.

## Figures and Tables

**Table I:** Characteristics of nine cases of diaphragmatic hernia following hepatic RFA.

Author (ref)	Year	Age, years	Gender	Tumor size (cm)	Location	Time from RFA to DH (months)	Treatment
Koda M *et al*(14)	2003	61	F	2.5	S8	13	Surgical repair
				1	S8		
				1.5	S6		
Shibuya A *et al*(15)	2006	72	M	2.8	S4 and S8	34 (18 months following repeated RFA)	Surgical repair
di Francesco F *et al*(16)	2008	49	M	5.4	Right lobe	15	Surgical repair
Nawa T *et al*(17)	2010	50	M	NA	S8	20	Surgical repair (LS)
Pan WD *et al*(18)	2010	37	M	NA	S4	5	Surgical repair (LS)
Boissier F *et al*(19)	2011	65	F	NA	S7	7	Surgical repair and colectomy
					S5	1	
Singh M *et al*(20)	2011	46	F	1.5	S2 and S3	19	Surgical repair (LS)
				1.5	S5 and S8		
Yamagami T *et al*(21)	2011	71	F	2.38	S7	9	Conservative treatment
Present case	2011	61	F	1.5	S8	12	Surgical repair and colectomy

RFA, radiofrequency ablation; DH, diaphragmatic hernia; F, female; M, male; NA, not available; LS, laparoscope.
